# Differential DNA methylation of potassium channel KCa3.1 and immune signalling pathways is associated with infant immune responses following BCG vaccination

**DOI:** 10.1038/s41598-018-31537-9

**Published:** 2018-08-30

**Authors:** Mateusz Hasso-Agopsowicz, Thomas J. Scriba, Willem A. Hanekom, Hazel M. Dockrell, Steven G. Smith

**Affiliations:** 1 Department of Immunology and Infection, Faculty of Infectious and Tropical Diseases, London School of Hygiene and Tropical Medicine, Keppel Street, London, WC1E 7HT United Kingdom; 2 South African Tuberculosis Vaccine Initiative and Institute of Infectious Disease and Molecular Medicine, Division of Immunology, Department of Pathology, University of Cape Town, Institute of Infectious Diseases and Molecular Medicine, Rondebosch, 7701 Cape Town South Africa

## Abstract

Bacillus Calmette–Guérin (BCG) is the only licensed vaccine for tuberculosis (TB) and induces highly variable protection against pulmonary disease in different countries. We hypothesised that DNA methylation is one of the molecular mechanisms driving variability in BCG-induced immune responses. DNA methylation in peripheral blood mononuclear cells (PBMC) from BCG vaccinated infants was measured and comparisons made between low and high BCG-specific cytokine responders. We found 318 genes and 67 pathways with distinct patterns of DNA methylation, including immune pathways, e.g. for T cell activation, that are known to directly affect immune responses. We also highlight signalling pathways that could indirectly affect the BCG-induced immune response: potassium and calcium channel, muscarinic acetylcholine receptor, G Protein coupled receptor (GPCR), glutamate signalling and WNT pathways. This study suggests that in addition to immune pathways, cellular processes drive vaccine-induced immune responses. Our results highlight mechanisms that require consideration when designing new TB vaccines.

## Introduction

*Mycobacterium bovis* Bacillus Calmette–Guérin (BCG) is the only licensed vaccine against tuberculosis (TB). BCG protects against severe childhood cases of miliary TB and tuberculous meningitis^1^. However, the protection afforded by the vaccine against pulmonary TB varies across geographical regions with studies reporting vaccine efficacy ranging from 0% in India^2^ to 80% in the United Kingdom (UK)^3^. Reasons for these variations remain unknown. Possible explanations include comorbidities^4^, pre-exposure to environmental mycobacteria^5^, and genetics^6^. Previous studies in Malawi^7^, The Gambia^8^, Indonesia^9^ and the UK^8,10^ have shown disparate immune responses across these populations, with a dominant production of IFNγ in the UK and greater production of T helper (Th)2 cytokines in Malawi and The Gambia^10^. Multiple efforts are being undertaken to identify correlates of protection following BCG vaccination^11,12^. The importance of IFNγ has been highlighted in numerous studies^8,13–16^, however, the Th1 boosting candidate TB vaccine MVA85A (Modified Vaccinia virus Ankara expressing Ag85A from *M. tuberculosis*) failed to improve on protection afforded by BCG in vaccinated infants^17^; a large South African study did not find that BCG specific Th1 response correlated with risk of TB disease^18^; however further analysis of the MVA85A clinical trial showed that the frequencies of cells producing BCG-specific IFNγ was associated with a reduced risk of developing disease^16^.

Another unknown factor in the immune response to the BCG vaccine is the molecular mechanism that drives these immune responses. Recent studies highlight the role of transcriptomics^19–21^ as means of identifying the key mechanisms and the importance of measuring RNA profiles in vaccine trials. Epigenetics, a mechanism known to play a role in regulation of gene expression, is another mechanism of growing importance. Specifically, DNA methylation of CpG dinucleotides in mammals is known to regulate gene expression and subsequent protein production^22,23^. *In-vitro* methylation of herpes thymidine kinase (tk) genes resulted in *in-vivo* downregulation of gene expression^24^ and methylation of the O^6^-methylguanine-DNA-methyltransferase (MGMT) gene was negatively correlated with its protein concentration in humans^25^. In the context of immune responses, two recent studies have examined the role of epigenetic regulation, gene expression and protein production in responses to Hepatitis B^26^ and Influenza^27^ vaccines. Several differentially methylated genes were found between low and high immune responders to Hep B vaccine and DNA methylation was correlated with gene expression and protein levels for multiple genes following influenza vaccination. Lastly, recent evidence shows that DNA methylation of macrophages is correlated with anti-mycobacterial activity in BCG vaccinated participants^28^.

We have examined whether the DNA methylation profile of peripheral blood mononuclear cells (PBMC) in BCG vaccinated infants is associated with the magnitude of BCG specific immune responses. We found novel pathways and genes that were differentially methylated between high and low BCG responders amongst South African infants who received BCG vaccination at birth. This knowledge will allow us to understand molecular mechanisms that drive vaccine-induced immune responses, paving the way to design better and more effective TB vaccines.

## Results

### Sample collection and processing

We used 60 archived frozen PBMC samples from a previous study^18^. 36.7% were female, 83.3% Cape Mixed Ancestry, 13.3% Black African and 3.3% Asian. All participants received the Japanese BCG vaccine at birth and their blood was collected at 10 weeks post vaccination.

### An increase in cytokine production after BCG stimulation is not correlated with cell phenotype

**Figure 1:**
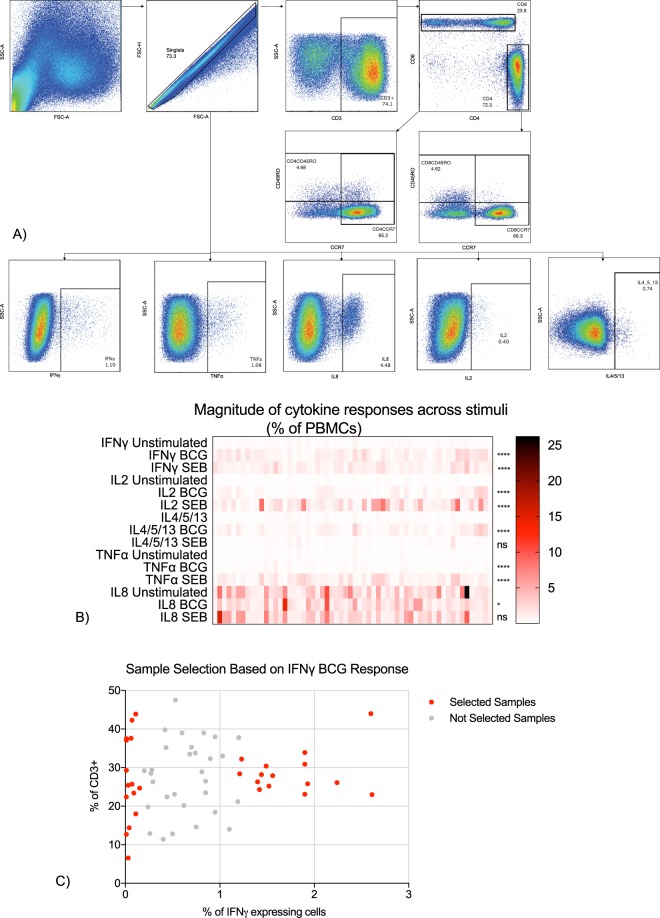
The immune response to the BCG vaccine was used to select samples for analysis. (**A**) The FACS gating strategy used to measure all immune responses. Singlet cells and CD3^+^ populations were chosen to measure intracellular cytokine responses as well as phenotypic markers such as CD3^+^, CD3^+^CD4^+^, CD3^+^CD8^+^, CD3^+^CD4^+^CCR7^+^, CD3^+^CD4^+^CD45RO^+^. (**B**) The magnitude of immune responses of PBMCs after 12 hours stimulation with BCG and SEB (% of PBMCs). Each column represents one donor sample. Stimulated samples were compared to non-stimulated with Wilcoxon paired t test. N = 60; ns = not significant; *p < 0.05; ****p < 0.0001 (**C**) A magnitude of IFNγ BCG responses when stratified by origin (CD3+ population). Fifteen lowest (non-responders) and fifteen highest (responders) IFNγ BCG producers (red) were selected for the DNA methylation analysis. Samples coloured in grey were not selected. N = 60.

To investigate the magnitude of immune responses to the BCG vaccine, we measured the cytokine production after stimulation with BCG (SSI strain, 1.2 × 10^6^ organisms/ml) or Staphylococcus Enterotoxin B (SEB) using an intracellular cytokine staining (ICS) assay. We looked at cytokine responses in the PBMC population (see gating strategy in Fig. 1A). There was a consistent increase in the production of all cytokines after BCG stimulation and IFNγ, TNFα, IL2 but not IL8 and IL4/5/13 after SEB stimulation (Fig. 1B). We examined whether the observed magnitude of immune responses was due to changes in T cell frequency and conducted a Spearman correlation analysis of frequencies of IFNγ-expressing cells following BCG stimulation with other immune parameters. The frequencies of BCG-specific IFNγ cells (hereafter called IFNγ BCG) were correlated with IL4/5/13, IL2 and IL8 secreted following stimulation with BCG but not with any T cell population measured (Table S1). This indicates that in this assay, the BCG induced IFNγ production is not correlated with T cell and lymphocyte composition. All samples were stratified based on their IFNγ BCG production and 15 highest (IFNγ Low) and 15 lowest (IFNγ High) IFNγ BCG producers were selected for the analysis of DNA methylation (Fig. 1C).

### Differential methylation of 318 genes between low and high BCG cytokine respondents

**Figure 2:**
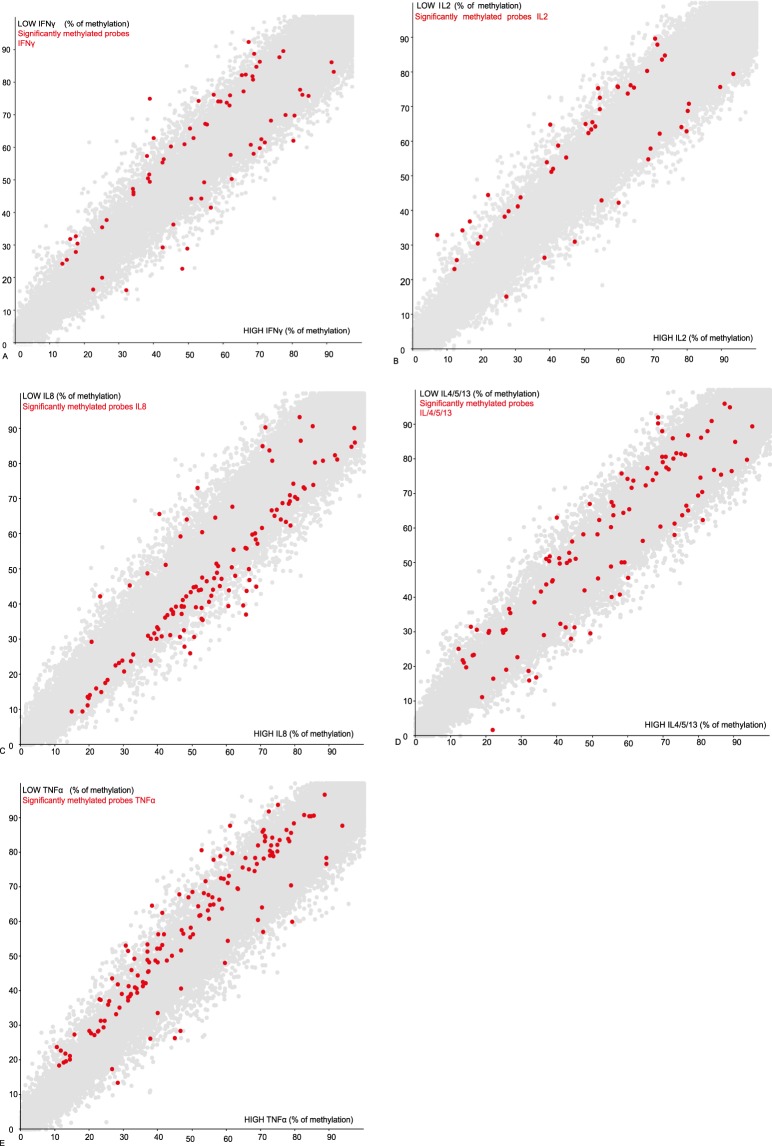
A scatter plot of all probes of which methylation was compared between a high and low cytokine responder group. Each dot represents a probe of which % methylation is measured in a high cytokine group (X axis) and low cytokine group (Y axis). Red dots represent probes which are significantly differentially methylated. BCG specific low and high cytokine groups that were compared included: IFNγ (**A**), IL2 (**B**), IL8 (**C**), IL4/5/13 (**D**), and TNFα (**E**).

A total of 30 samples were selected for the DNA methylation analysis. These were stratified based on their BCG specific immune responses forming low vs. high groups for five cytokines: IFNγ; IL4/5/13; IL2; TNFα; and IL8. For each cytokine, DNA methylation of low responders was compared to high responders using logistic regression with a minimum difference in methylation of 5%. The analysis showed several probes to be differentially methylated between low and high groups of: IFNγ (70), IL4/5/13 (108), IL2 (50), TNFα (146), and IL8 (122) (Fig. 2). A gene feature report highlighted 318 genes (Table 1) that span the differentially methylated probes. Several novel genes of which levels of methylation can indirectly affect the magnitude of BCG immune responses were identified. Notably, the ZFP57 gene was found to be differentially methylated in all cytokine low and high groups comparisons. ZFP57 is a transcriptional regulator of gene imprinting and it acts by controlling DNA methylation during the earliest stages of multicellular development^29,30^. Another gene candidate KCa3.1 is a protein that forms the voltage-independent potassium channel and was found to be differentially methylated across 4 cytokine low and high groups (IFNγ, IL2, IL4/5/13 and IL8). Kca3.1 regulates calcium influx and can influence TH1 and TH2 development^31^. We also found ERICH-1 to be differentially methylated across cytokine low and high groups. ERICH-1 encodes the a glutamate rich 1 protein and glutamate is known to regulate Ca^2+^ and K^+^ efflux thereby affecting T cell activation^32^.

**Table 1:** A summary of all genes that were differentially methylated across all low and high cytokine groups.

Name of Cytokine Pairs	Total Number of Differentially Methylated Genes	Name of Genes
IFNγ IL2 IL4/5/13 IL8 TNFα	1	ZFP57
IFNγ IL2 IL4/5/13 IL8	2	PTPRN2 KCNN4
IFNγ IL2 IL4/5/13 TNFα	4	ERICH1 GPR124 RP5-1029F21.3 COLEC11
IL2 IL4/5/13 IL8 TNFα	2	CTC-281F24.1 MIR4520-1
IFNγ IL2 IL4/5/13	5	RP11-609L3.1 ZMIZ1 AURKC NEURL1B SEPT7P3
IFNγ IL2 TNFα	1	DNAH1
IFNγ IL4/5/13 IL8	1	AZU1
IFNγ IL4/5/13 TNFα	3	LMTK3 LY6K ANO8
IL2 IL8 TNFα	1	CRYGEP
IL2 IL4/5/13 TNFα	1	DTX1
IL4/5/13 IL8 TNFα	2	EHBP1L1 SIPA1
IFNγ IL2	2	PRSS36 MVB12B
IFNγ IL8	1	GCSAML
IFNγ IL4/5/13	17	TTN DEAF1 CARS2 RP11-108K14.12 HELZ2 RGS3 COL1A1 MTHFR GRK1 DUSP15 CYP2E1 FIP1L1 LPIN1 TTN-AS1 C1orf167 FBXO41 CMTM7
IFNγ TNFα	1	LINC00539
IL2 IL8	1	HCN2
IL2 IL4/5/13	3	PDIA3P2 CATSPER2 KCNAB1
IL2 TNFα	2	PROSER3 MAN1C1
IL8 TNFα	3	CUX1 PLEC SLC43A2
IL4/5/13 TNFα	9	PKNOX2 HNRNPH1 AC010907.2 RP11-687M24.8 MAST4 LHX6 ADARB2 AC096649.3 NLGN2
IFNγ	13	MED15P5 NTM CTD-3080P12.3 PIAS4 SPG7 TIMP2 CAMSAP3 SOGA3 CD9 MALRD1 PXDN NTNG2 RP11-599J14.2
IL2	15	RP4-724E13.2 TGFBR3 RP11-452L6.8 COX6A2 CARD11 NINJ2 SNRPN MAP1LC3BP1 RP11-218M22.1 C20orf96 ANK1 FANCC MEGF6 RP4-559A3.7 LEFTY1
IL8	88	RN7SL646P FAM222B ZFP41 PBX4 LINC01169 KCNQ1OT1 TRPV2 EDARADD UMODL1-AS1 DTNB bP-21264C1.2 RP11-216L13.18 KIAA1161 CDH23 ZNF473 LINC01266 COL23A1 AC011850.2 FLJ26850 RP11-394I13.3 LINC00467 SOX10 MSL3P1 AC018688.1 HIST1H3E FAM217B 5_8S_rRNA RNMTL1 RAI1 RELT LRRC24 NFATC1 UNC93B1 ZNF790-AS1 EMC9 ITPR1 POU5F1 EGOT UMODL1 MIR4508 AC005481.5 VOPP1 AC005538.3 ATP8B3 POLR2F MIR6820 SDK1 MIR138-2 FRRS1 MIR6813 WDR60 COL5A1 CTD-2126E3.3 THSD4 OPCML TMPRSS12 RABL6 RP11-133L14.5 PROSER2-AS1 PIK3R2 PRSS21 RYR1 CLEC9A CTD-2269E23.4 MAN2B1 ARHGEF10 GLI4 RP11-497G19.1 ATG16L2 LZTS3 WDR20 SH3GL1 EXD3 AMOTL2 LRRC14 MROH1 CTD-2192J16.22 TARID CFD TNFRSF11A MMP17 RGS19 KCNQ1 C8orf82 RNF165 LOXL1 LINC01312 SYCP2
IL4/5/13	41	TPM4P1 GNAL AC131097.4 RP11-244F12.1 RP11-867G23.12 NBPF13P RNVU1-8 RIMBP2 LINC01237 ZNF718 RP11-680G24.4 TAF1C TBCE PHACTR3 RP11-662M24.2 RP11-452D2.2 RP11-545A16.3 HLA-DRB9 PRDM15 KCNN3 PRDM16 DRAXIN ARID3A SNRNP27 COL18A1 RP11-1260E13.1 RPH3AL RP3-522J7.6 AP001347.6 LRRC37A6P PRDM7 MYBPC2 PNPLA6 AC147651.1 PDXDC1 SRC NYAP2 ADAMTS12 ANKRD20A18P C5orf17 RIN1
TNFα	98	GABRB3 MKRN3 PRKAR1B PAX8-AS1 FAM189A1 RNA5-8S5 ACOT2 WT1 ACVR1B LINC00839 AOX3P PRDM8 LDLRAD2 CHCHD3 FBXO16 PEBP4 MARK4 CTU1 HLA-DPA1 ATP5J2 RPL13A MEST RASAL2 PRAM1 PRKCQ GNAI2 RP11-127L20.3 RP11-726G23.10 BRSK1 BCAN CROCC GABRA5 LINC00887 PTCD1 SDF4 FNDC3B RPL13P12 CYGB NCOR2 CLIP2 GALNT9 BCR AOX2P OPRL1 RP11-377G16.2 FLT3LG CTD-3148I10.9 CPT1A RP11-54O7.17 RPS6KA4 RP11-284F21.7 PIGQ RP11-492E3.2 PRCD AC093609.1 HLA-DPB1 SEMA6B CH507-513H4.1 UHRF1 CTD-3148I10.15 SEMA3B ZNF331 GEMIN7 MPPED1 ERICH1-AS1 RP5-906A24.1 PPAPDC3 MGRN1 ZNF395 FLT4 ITPRIP RP11-426C22.5 KAZN MST1L PSPN ITFG2 ARL5C RP11-137H2.4 FBXW4P1 PAX8 ALKBH7 C17orf98 RBM19 NOS3 CCNYL2 RP11-273G15.2 HTRA4 RP11-231D20.2 RP11-65I12.1 SCARF1 ITGAX RP11-230C9.1 PPP1R37 RP11-56P9.10 OSBPL5 ATP5J2-PTCD1 CBFA2T3 PLEKHA2

Each row shows single or multiple genes that were differentially methylated across one or multiple cytokine groups. n = 30.

### Hierarchical cluster analysis reveals clusters of differentially methylated genes

**Figure 3:**
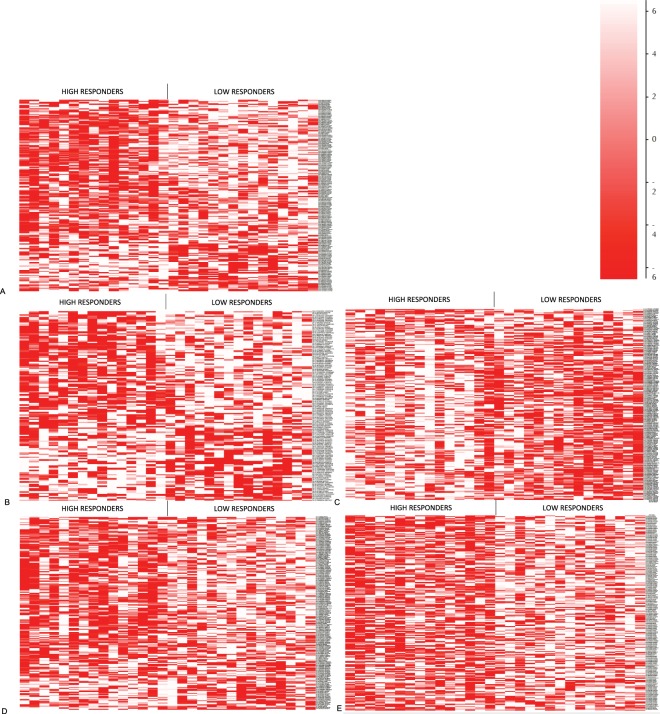
A hierarchical per probe normalised clustering analysis of the DNA methylation profile between high and low IFNγ (**A**), IL2 (**B**), IL8 (**C**), IL4/5/13 (**D**) and TNFα (**E**) responder groups. First fifteen columns represent a methylation of probes in cytokine high responder group and last fifteen columns represent a methylation of probes in the low responder group. Each horizontal line represents a differentially methylated probe. n = 30.

We conducted a t-distributed stochastic neighbour embedding (tSNE) analysis of gene methylation for the 30 examined samples, however this did not show a clear separation of IFNγ low and IFNγ high nor the other cytokine low and high groups (data not shown). This indicates subtle differences in DNA methylation between cytokine low and high groups, suggesting that there might also be other mechanisms that drive immune responses to the BCG vaccine. We then proceeded with a hierarchical cluster analysis of the differentially methylated probes when stratified by cytokine respondents. We saw visible differences in cluster formation for all the cytokine groups measured (Fig. 3).

### Differentially methylated genes are part of immune and cellular processes pathways

We examined biological functions of the differentially methylated genes for all the cytokines using the Panther Pathway Analysis (PPA). Differentially methylated genes are included in 67 pathways that are involved in multiple biological processes including immune functions (2.8%), metabolic (20.8%) and cellular (25.3%) processes and other processes (Table 2). Several pathways of the immune system could explain the polarisation of cytokine responses. Notably, T cell activation, interferon gamma signalling, interleukin and JAK/STAT signalling pathways were all involved in the regulation of cytokine responses. B cell activation, TGF-beta and chemokine and cytokine mediated inflammation were also highlighted as pathways containing genes that were differentially methylated. A large proportion of highlighted pathways regulate cellular and metabolic processes. We found differentially methylated genes that are part of the G-protein signalling pathways, which are known to regulate T cell migration and activation^33^. We also found 6 differentially methylated genes in the Wnt pathway, which is known to regulate IL12 signalling in antigen presenting cells (APCs)^34^. Lastly, we highlight the muscarinic acetylcholine receptor signalling pathway (mAChRs), which can mediate cytokine production^35^. By regulating the immune signalling and cellular processes these pathways can directly and indirectly affect the magnitude of the BCG induced immune response that is observed in our samples.

**Table 2:** An overview of biological processes and selected pathways of the differentially methylated genes when stratified by low and high cytokine groups.

Name of the Biological Process	Number of Genes Involved	% of Total Genes
**Biological Processes Of Differentially Methylated Genes**		
Cellular Process	80	25.3
Metabolic Process	66	20.8
Response To Stimulus	34	10.8
Biological Regulation	30	9.4
Developmental Process	30	9.4
Multicellular Organismal Process	25	7.8
Localization	18	5.6
Cellular Component Organization Or Biogenesis	13	4.2
Immune System Process	9	2.8
Biological Adhesion	6	1.9
Locomotion	4	1.1
Reproduction	3	0.8
**Pathway Name**	**Number of Genes Involved**	**% of Total Genes**
**Highlighted Pathways of Differentially Methylated Genes**		
Heterotrimeric G-Protein Signaling Pathway-Gi Alpha And Gs Alpha Mediated Pathway	7	4.2
Integrin Signaling Pathway	6	3.6
Heterotrimeric G-Protein Signaling Pathway-Gq Alpha And Go Alpha Mediated Pathway	6	3.6
Wnt Signalling Pathway	6	3.6
T Cell Activation	5	3.0
Inflammation Mediated By Chemokine And Cytokine Signaling Pathway	3	1.8
Muscarinic Acetylcholine Receptor 1 And 3 Signaling Pathway	3	1.8

Each row shows a biological process or a pathway and a number of differentially methylated genes associated with it.

## Discussion

BCG vaccination induces variable immunity across populations^36,37^ translating to an inconsistent protection against pulmonary tuberculosis^1^. This study aimed to answer whether epigenetic modifications, specifically DNA methylation, could be one of the molecular mechanisms that drive the observed disparate immune responses.

We first measured the magnitude of immune responses induced after the BCG vaccination. We chose to include all cells in the PBMC population as the DNA used for the methylation analysis was extracted from matching vials of PBMC. Similar to the previous study^18^, we saw an increase of all the measured cytokines following BCG stimulation *in vitro*. The magnitude of immune responses for IFNγ ranged from 0% to 2.61% measured as the frequency of cytokine producing PBMCs. As expected, BCG and SEB stimulation decreased the expression of CD3 protein and had no effect on the expression of CD4 and CD8 proteins (data not shown) and SEB, an inflammatory superantigen^38^, failed to increase the production of TH2 cytokines and IL8. CD4+ and CD8+ T cells are known to be the main producers of IFNγ^39^, however, we saw that none of the T cell phenotypes measured correlated with levels of BCG induced IFNγ (Table S1). This indicates that the magnitude of immune responses is independent of cellular T cell composition and is likely driven by molecular mechanisms within individual cells. The frequencies of BCG-induced IFNγ had a strong positive correlation with IL2 and IL4/5/13, and a weak negative correlation with IL8. IL2 is known to activate T cells, which are one of the main IFNγ producers^40^, and the BCG vaccine was shown to previously induce both TH1 and TH2 cytokines^7,9^. We cannot exclude that changes in cell numbers of Natural Killer (NK) cells and monocytes between groups could explain observed differences in cytokine levels. This could be addressed by analysing DNA methylation patterns in purified cell populations, which due to a limited sample size was beyond the scope of the project.

Having measured the immune responses and selected samples for further analysis, we measured DNA methylation and stratified samples by high and low cytokine responses. We found probes that were differentially methylated between high and low cytokine responders and identified the 318 corresponding genes. Interestingly, genes that were differentially methylated in many of the cytokine groups were involved in potassium, calcium and neurotransmitter signalling.

KCNN4 or Kca3.1, Potassium Calcium-Activated Channel Subfamily N Member 4, was found to be differentially methylated in IFNγ, IL2, IL4/5/13, and IL8 high and low groups. The Kca3.1 channel is a voltage-independent potassium channel, which is activated by an increase in intracellular calcium, resulting in membrane hyperpolarization and continuous calcium influx^31^. Calcium regulates multiple cellular processes. A prolonged contact between a CD4^+^ T cell and an antigen presenting cell (APC) results in an increase in intracellular Ca^2+^ levels. The main sources of Ca^2+^ in T cells are store-operated calcium channels (SOCE) and calcium-release-activated calcium (CRAC)^41–44^. A SOCE and CRAC dependent release of calcium inside a CD4+ T cells changes gene expression, and thus cytokine production, drives the differentiation of naïve T cells to TH1 or TH2 cells, and the development of immature T cells^41^. A strong Ca^2+^ signal favours TH1 differentiation^45^ whereas weak Ca^2+^ release skews cells to a TH2 phenotype^46–48^. Kca3.1 can thus affect T cell activation by maintaining a negative membrane potential and continuing Ca^2+^ release via CRAC channels^31^.

ERICH1, which encodes the glutamate rich 1 protein, was found to be differentially methylated in 4 out of 5 low and high cytokine groups. Glutamate is an excitory neurotransmitter of the nervous system, which is critical for brain development and function and plays a signalling role in peripheral organs, including T lymphocytes^32,49^. It can be produced by several cells of the immune system including T cells, dendritic cells and others. Glutamate mediates intracellular Ca^2+^ fluxes and outward K^+^ flows thereby affecting adhesion, migration, proliferation, survival, activation and metabolism of T cells^32,49,50^. A differential methylation of ERICH1 could therefore lead to varied intracellular glutamate levels affecting the immune response to the BCG vaccine.

To look more broadly at differentially methylated genes, we investigated the array of pathways which contained differentially methylated genes. As expected, we found several immune pathways: T cell activation, JAK/STAT signalling, Interleukin signalling, Interferon-gamma signalling, inflammation mediated by chemokine and cytokine signalling, integrin signalling, TGF-beta signalling, B cell activation. All these pathways could contribute to the underlying differences observed in the cytokine response to the BCG vaccine. Interestingly, we also found a large proportion of pathways that were involved in metabolic and cellular processes. Several neurotransmitter mediating pathways were highlighted.

Muscarinic acetylcholine receptor signalling pathway signals via muscarinic acetylcholine receptors (mAChRs), which is made up of five types (M_1_–M_5_) of class A G protein-coupled receptors (GPCRs)^51^. Activation of T cells with PHA and PMA upregulates the expression of mAChRs^52,53^; a stimulation via CD11a upregulates M_5_ gene expression^52^ suggesting an increase in cholinergic transmission following T cell activation; a stimulation of mAChRs in PBMC upregulated the IL2 production^35^; and M_1_-M_5_ knockout mice produced lower levels of OVA Specific IgG1, TNFα and IL-6^54^.

Seven genes in the GPCRs signalling pathway were differentially methylated across low and high cytokine groups. G proteins act as molecular switches to control downstream effector molecules including chemokines and chemokine receptors^33^. In immune cells, G proteins impact signal transduction and affect survival, proliferation, differentiation and cell migration and recent evidence suggests non-canonical GPCR signalling in immune cells^33,55^.

The WNT pathway, of which 6 genes were differentially methylated, is known to indirectly influence the immune system. There are 19 WNT genes in the human genome and they all encode lipid-modified secreted glycoproteins. There is numerous evidence that show how the WNT proteins affect the immune system: WNT1 and WNT4 deficient mice have impaired T and B cell development^56,57^; WNT proteins are crucial for the initial proliferation of thymocytes before β-selection^58^; the overexpression of WNT signalling increases the survival of CD4^+^CD25^+^ regulatory T cells (Tregs)^59^; and WNT signalling in APCs results in increased IL12 production and subsequent TH1 differentiation^34^. Variation in methylation of WNT genes could therefore lead to changes in WNT proteins, ultimately leading to mediating the immune response to the BCG vaccine.

A report by Verma *et al*.^28^ has recently investigated the effect of BCG vaccination on the DNA methylation of PBMCs *in vivo*. They found an enrichment of the DNA methylation in genes of immune pathways, including T cell activation, after the BCG vaccine vs. before vaccination. Their study shows that several non-immune pathways such as regulation of biological processes are enriched however these processes were not discussed in the report. It is important to highlight notable differences between the current study and that of Verma *et al*. We investigated the preceding methylation patterns induced by BCG vaccination and how they regulate the immune response to BCG stimulation in infants whereas the Verma *et al*. study investigated how BCG vaccination modulates the DNA methylation of PBMCs in adults.

Hierarchical cluster analysis of DNA methylation revealed visible differences in cluster formation between high and low IFNγ groups as well as other cytokines but tSNE analysis failed to separate infants based on their DNA methylation patterns. These findings suggest that DNA methylation is probably not the main mechanism to regulate the immune response to the BCG vaccine and other epigenetic mechanisms might play a role. Histone modifications and microRNA are other types of epigenetic modifications that could explain differences in observed immune responses. Histone modifications are known to control transcriptional profile of memory lymphocytes thereby shaping their function^60^. They also regulate mechanisms of adaptive features of trained immunity^61^. microRNAs have shown to play a role in modulation of inflammatory responses^62^.

We acknowledge that the observed differences in DNA methylation patterns may not be specific to the BCG vaccine responses and may in fact, represent a regulation of general vaccine induced immune responses. However, it is important to highlight that in a context of this study, cytokine levels were measured after BCG stimulation, hence they are BCG specific. Thus, the differences in cytokine levels and the subsequent stratification to low and high cytokine responding groups is BCG specific. The DNA methylation differences observed between these groups are associated with BCG specific differences in cytokine responses, however we cannot exclude that they may also be associated with immune responses to other vaccines.

Taken together, the data reported here identified genes and pathways that were differentially methylated in PBMC from infants with high or low cytokine responses to BCG vaccination. As expected we observed a differential methylation of multiple immune pathways that could directly influence the disparate immune responses to the BCG vaccine. Unexpectedly, we identified several genes and pathways that could indirectly affect the BCG specific cytokine production. These findings suggest that in addition to immune pathways, vaccine induced immune responses could be modulated by molecular mechanisms and mediators that regulate cellular processes such as glutamate, flux of potassium and calcium through membrane channels, GPCRs and mACHRs signalling and the WNT pathway. Studies should focus on investigating the role of methylation as well as other epigenetic marks in the regulation of the genes and pathways in question as well as measuring differences in gene expression when stratified by high and low cytokine responses. This knowledge will allow us to understand the molecular mechanisms that drive vaccine-induced immune responses, paving a way to design better and more effective TB vaccines.

## Methods

The datasets generated during and/or analysed during the current study are available from the corresponding author on reasonable request.

### Study participants

We retrieved blood samples collected from a subset of 10 week old infants who were enrolled into a large study of BCG vaccination at the South African Vaccine Initiative (SATVI) field site in the Worcester area, near Cape Town, South Africa (Hawkridge *et al*., BMJ 2008^18^. Parents All participants were vaccinated with BCG at birth and samples were collected 10 weeks after birth. Participants were excluded from the study if any of the exclusion criteria were met: infant not immunised with BCG within 24 hours; mother infected with human immunodeficiency virus (HIV); chronic and acute disease in the infant at the time of enrolment; clinical anaemia in the infant; significant perinatal complications in the infant; and contact with any person with TB disease or anyone who was coughing. Participants were followed for two years to observe the development of TB disease. Only healthy infants who did not develop TB disease were selected for this study. Parents gave consent for their infants to participate in the study. The study was conducted according to the U.S. Department of Health and Human Services and Good Clinical Practice guidelines, and included protocol approval by the University of Cape Town Research Ethics Committee and written informed consent from the parent or legal guardian. The study also received ethical approval from the Ethics Committee of the London School of Hygiene and Tropical Medicine (LSHTM, #8720).

### Cell separation, processing and stimulation

The methods below have been previously published in other studies^18,63^. Heparinized blood samples were collected from infants. Peripheral blood mononuclear cells were isolated using the density gradient centrifugation. The remaining 1 ml of blood was incubated with BCG (SSI, 1.2 × 10^6^ organisms/ml), medium alone, and staphylococcal enterotoxin B (10 μg/ml; Sigma-Aldrich) (SEB). The co-stimulatory antibodies, anti-CD28 and anti-CD49d antibodies (1 μg/ml each; BD Biosciences, San Jose, CA) were added to all conditions. Samples were incubated for 7 hours at 37 °C. Later, Brefeldin-A was added and samples were incubated for another 5 hours. Red blood cells were lysed and white cells fixed using FACS Lysing Solution (BD Biosciences). Cells were collected, fixed and cryopreserved as described elsewhere^63^. Thus, for each participant non-stimulated PBMC sample and three stimulated and fixed whole blood samples were available.

### Intracellular cytokine staining

Previously stimulated samples were thawed and washed in FACS buffer (PBS, 5% FBS v/v, 0.05% sodium azide w/v) and stained for 30 minutes at 4 °C in the dark, with a cocktail of surface marker antibodies: CD3-BV650 (clone SK7), CD4-BV605 (clone S3.5), CCR7-PECF594 (clone 150503), CD45RO-APC-H7 (clone UCHL1), (all BD Bioscience) and CD8-BV570 (clone RPA-T8; BioLegend). Samples were then permeabilised using the Cytoperm/Cytofix (BD Pharmingen) solution for 20 mins. After permeabilisation, cells were stained for 30 minutes at room temperature in the dark with a cocktail of intracellular cytokine antibodies: TNFα-PE-Cy7 (clone Mab11), IFNγ-V450 (clone B27), IL-2-FITC (clone MQ1-17H12), (BD Bioscience), IL-4-PE (clone 3010.211; FastImmune), IL-5-PE (clone TRFK5), IL-13-PE (JES10-5A2) and IL-8-APC (clone E8A1), (BioLegend). IL4, 5 and 13 were collected in one PE channel. After staining, samples were washed and stored at 4 °C in the dark to be acquired within 24 hours using BD LSRII Flow Cytometer and acquiring a minimum of 200,00 events.

### Sample selection and DNA isolation

Samples were stratified based on the results of the ICS assay and the BCG specific IFNγ response. Corresponding PBMC samples of the 15 lowest (hereafter called IFNγ Low) and 15 highest IFNγ (hereafter called IFNγ High) respondents were selected for the DNA methylation analysis. The selected 30 samples were later stratified based on their IL2, IL8, IL4/5/13 and TNFα responses effectively forming 5 low and high cytokine groups. DNA was extracted using the Dneasy Blood & Tissue Kit (Qiagen). The quality of the DNA was checked with NanoDrop (Thermo Fisher Scientific) and the concentration measured with Qubit dsDNA HS Assay Kit (Thermo Fisher Scientific).

### Measurement of the DNA methylation

The DNA methylation was examined using a reduced representation bisulphite sequencing (RRBS) method and with the Premium Reduced Representation Bisulphite Sequencing Kit (Diagenode) according to manufacturer’s instructions.

### DNA quality control and MiSeq sequencing

DNA concentration was measured using the Qubit dsDNA HS Assay Kit and size examined with the High Sensitivity DNA Analysis Kit (Genomics Agilent) using Bioanalyzer 2100 (Genomics Agilent). DNA was denatured with 0.2 M NaOH and its concentration was adjusted to 16pM. Samples were sequenced with the MiSeq Reagent Kit v3 (Illumina) at 51 cycles per run and single end reads. A 12.5 pM PhiX v3 library (Illumina) was added for a positive control.

### Data processing

FASTA files were generated as the DNA sequencing output. Sequences were trimmed using the TrimGalore (v0.4.3) and Cutadapt software discarding all reads below 30 Phred quality score. The quality of samples was measured using the FastQC software (v0.11.5). A bisulphite treated human genome reference sequence was prepared using the GrCh38p7 assembly and Bismark Genome Preparation (v0.16.3) and Bowtie2 (v2.2.9) software. Samples were mapped to the reference genome using Bismark (v0.16.3) and Bowtie2. The DNA methylation was extracted using Bismark Methylation Extractor (v0.16.3).

### Data analysis: DNA methylation

The methylation of DNA sequences was analysed using SeqMonk (v1.38.2) and Rstudio (1.0.44) software. Contig methylation probes were generated with a 10-fold depth cut off, ignoring duplicate reads and merging probes closer that 500 bp. Probes were quantified using read count quantitation and filtered to include probes measured at least 10 times. Chromosomes X, Y and any mitochondrial DNA were removed and probes were quantitated using bisulphite methylation pipeline embedded in the SeqMonk software. Based on the previous ICS responses of whole blood samples, DNA samples were then separated into two groups: IFNγ low & IFNγ high and a logistic regression with a P-value cut-off of 0.05 and multiple testing correction was run. The logistic regression test was then performed on other cytokine groups: IL4/5/13 low & IL4/5/13 high; IL2 low & IL2 high; TNFα low & TNFα high; and IL8 low and IL8 high. Genes that were differentially methylated were visualised using SeqMonk, Panther Pathway Analysis and Cytospace (v3.5.1).

### Data analysis: correlation of immune responses

We used StataSE (v15.0) software to measure whether the magnitude of the BCG induced immune response is cell population dependent. Frequencies of BCG specific cytokines (TNFα, IFNγ, IL2, IL8 and IL4/5/13) and cell phenotypes (CD3 + CD4 + CCR7 + , CD3 + CD4 + CD45RO + , CD3 + , CD3 + CD4 + , CD3 + CD8 + , % of Lymphocytes) were correlated with the BCG specific IFNγ response using spearman correlation.

## Electronic supplementary material

Additional Results Information
