# Supplementary material for: Differential DNA methylation of potassium channel KCa3.1 and immune signalling pathways is associated with infant immune responses following BCG vaccination
Source: Sci Rep. 2018 Aug 30;8:13086. doi: 10.1038/s41598-018-31537-9 (PMC6117309; doi:10.1038/s41598-018-31537-9)
Supplement: Supplementary file 1 — Additional Results Information [file 41598_2018_31537_MOESM1_ESM.pdf]

Title

Differential DNA methylation of potassium channel KCa3.1 and immune signalling pathways is associated with infant immune responses following BCG vaccination

Authors/Affiliations

Mateusz Hasso-Agopsowicz,[1,*] Thomas J Scriba,[2] Willem Hanekom,[2] Hazel M Dockrell,[1] Steven G Smith[1,3,*]

[1]Department of Immunology and Infection, Faculty of Infectious and Tropical Diseases, London School of Hygiene and Tropical Medicine, Keppel Street, London, WC1E 7HT, United Kingdom

[2]South African Tuberculosis Vaccine Initiative and Institute of Infectious Disease and Molecular Medicine, Division of Immunology, Department of Pathology, University of Cape Town, Institute of Infectious Diseases and Molecular Medicine, Rondebosch 7701, Cape Town, South Africa

[3]Lead Contact

*Correspondence: mjagops@gmail.com (M.H.A), steven.smith@lshtm.ac.uk (S.S.)

Table S1: A spearman correlation analysis of IFNγ BCG levels with immune parameters. There is a strong correlation between IFNγ BCG and IL/4/5/13 BCG, IL2 BCG and a weak correlation with IL8 BCG. No other parameters were correlated with IFNγ BCg. n=60.

Immune Parameter	Correlation "R"	P value
IFNγ BCG	1.00	0.0000
IL4/5/13 BCG	0.53	0.0000
IL2 BCG	0.53	0.0000
IL8 BCG	-0.28	0.0313
CD3+CD4+CCR7+	-0.24	0.0614
CD3+CD4+CD45RO+	0.22	0.0959
TNFα BCG	0.21	0.1042
CD3+	0.11	0.3999
% of Lymphocytes	0.10	0.4340
CD3+CD4+	0.09	0.4867
CD3+CD8+	0.05	0.7093

Table S2: An overview of all pathways of the differentially methylated genes when stratified by low and high cytokine response. Each row shows a biological process or a pathway and a number of differentially methylated genes associated with it.

Pathway	Genes Involved	% of Total
Axon guidance mediated by netrin (P00009)	2	1.20%
Beta3 adrenergic receptor signalling pathway (P04379)	1	0.60%
Axon guidance mediated by Slit/Robo (P00008)	1	0.60%
Metabotropic glutamate receptor group III pathway (P00039)	2	1.20%
Beta2 adrenergic receptor signalling pathway (P04378)	2	1.20%
JAK/STAT signalling pathway (P00038)	1	0.60%
Beta1 adrenergic receptor signalling pathway (P04377)	2	1.20%
Apoptosis signalling pathway (P00006)	3	1.80%
5HT4 type receptor mediated signalling pathway (P04376)	1	0.60%
Angiogenesis (P00005)	7	4.20%
Interleukin signalling pathway (P00036)	1	0.60%
Alzheimer disease-presenting pathway (P00004)	1	0.60%
Interferon-gamma signalling pathway (P00035)	1	0.60%
5HT2 type receptor mediated signalling pathway (P04374)	2	1.20%

Alzheimer disease-amyloid secretase pathway (P00003)	2	1.20%
Integrin signalling pathway (P00034)	6	3.60%
5HT1 type receptor mediated signalling pathway (P04373)	1	0.60%
Alpha adrenergic receptor signalling pathway (P00002)	2	1.20%
Insulin/IGF pathway-protein kinase B signalling cascade (P00033)	1	0.60%
Insulin/IGF pathway-mitogen activated protein kinase kinase/MAP kinase cascade (P00032)	1	0.60%
Inflammation mediated by chemokine and cytokine signalling pathway (P00031)	3	1.80%
Hypoxia response via HIF activation (P00030)	1	0.60%
GABA-B receptor II signalling (P05731)	1	0.60%
Huntington disease (P00029)	2	1.20%
Heterotrimeric G-protein signalling pathway-rod outer segment phototransduction (P00028)	1	0.60%
p53 pathway (P00059)	1	0.60%
p53 pathway feedback loops 2 (P04398)	1	0.60%
Heterotrimeric G-protein signalling pathway-Gq alpha and Go alpha mediated pathway (P00027)	6	3.60%
Heterotrimeric G-protein signalling pathway-Gi alpha and Gs alpha mediated pathway (P00026)	7	4.20%
Wnt signalling pathway (P00057)	6	3.60%
Hedgehog signalling pathway (P00025)	1	0.60%
VEGF signalling pathway (P00056)	5	3.00%
Allantoin degradation (P02725)	1	0.60%
Transcription regulation by bZIP transcription factor (P00055)	3	1.80%
Thyrotropin-releasing hormone receptor signalling pathway (P04394)	2	1.20%
General transcription regulation (P00023)	2	1.20%
Ras Pathway (P04393)	1	0.60%
General transcription by RNA polymerase I (P00022)	1	0.60%
T cell activation (P00053)	5	3.00%
FGF signalling pathway (P00021)	3	1.80%
TGF-beta signalling pathway (P00052)	3	1.80%
Oxytocin receptor mediated signalling pathway (P04391)	2	1.20%
Endothelin signalling pathway (P00019)	7	4.20%
EGF receptor signalling pathway (P00018)	4	2.40%
p38 MAPK pathway (P05918)	1	0.60%
Parkinson disease (P00049)	1	0.60%

PI3 kinase pathway (P00048)	3	1.80%
Opioid proopiomelanocortin pathway (P05917)	1	0.60%
PDGF signalling pathway (P00047)	4	2.40%
Opioid prodynorphin pathway (P05916)	1	0.60%
Histamine H2 receptor mediated signalling pathway (P04386)	1	0.60%
Oxidative stress response (P00046)	2	1.20%
Opioid proenkephalin pathway (P05915)	1	0.60%
Histamine H1 receptor mediated signalling pathway (P04385)	3	1.80%
Notch signalling pathway (P00045)	1	0.60%
Cadherin signalling pathway (P00012)	2	1.20%
Enkephalin release (P05913)	2	1.20%
Muscarinic acetylcholine receptor 2 and 4 signalling pathway (P00043)	2	1.20%
Dopamine receptor mediated signalling pathway (P05912)	1	0.60%
Muscarinic acetylcholine receptor 1 and 3 signalling pathway (P00042)	3	1.80%
B cell activation (P00010)	3	1.80%
Angiotensin II-stimulated signalling through G proteins and beta-arrestin (P05911)	1	0.60%
Metabotropic glutamate receptor group I pathway (P00041)	2	1.20%
Cortocotropin releasing factor receptor signalling pathway (P04380)	1	0.60%
Metabotropic glutamate receptor group II pathway (P00040)	2	1.20%
CCKR signalling map (P06959)	6	3.60%
Gonadotropin-releasing hormone receptor pathway (P06664)	12	7.30%
